# Pregabalin prescribing patterns in Australian general practice, 2012–2018: a cross-sectional study

**DOI:** 10.3399/bjgpopen20X101120

**Published:** 2020-12-02

**Authors:** Andrea L Schaffer, Doreen Busingye, Kendal Chidwick, Jonathan Brett, Suzanne Blogg

**Affiliations:** 1 Centre for Big Data Research in Health, University of New South Wales, Sydney, New South Wales, Australia; 2 NPS Medicinewise, Strawberry Hills, New South Wales, Australia

**Keywords:** general practice, gabapentinoids, pregabalin, neuropathic pain, neuralgia, epidemiology

## Abstract

**Background:**

In 2013 pregabalin was subsidised by Australia’s Pharmaceutical Benefits Scheme (PBS) for neuropathic pain. Since the subsidy, pregabalin prescribing has been increasing in Australia and so has related harm. There are concerns it is being prescribed for indications other than neuropathic pain, which have little evidence of efficacy.

**Aim:**

To describe pregabalin prescribing in Australian general practice.

**Design & setting:**

A cross-sectional study of patients attending 445 general practice sites in the national MedicineInsight database from March 2012–February 2018.

**Method:**

The following aspects were calculated: the proportion of prescriptions that were for pregabalin per year; the prevalence of pain conditions in patients prescribed pregabalin; and same-day prescribing of pregabalin with opioids or benzodiazepines.

**Results:**

Prescribing increased from 13 per 10 000 to 104 per 10 000 prescriptions between 2012–2013 and 2017–2018. A total of 1 891 623 patients were identified of whom 114 123 (6.0%) were prescribed pregabalin; 49.7% (*n* = 56 772) had a recorded diagnosis of neuropathic pain. Among people prescribed pregabalin without a recorded diagnosis of neuropathic pain, 43.5% (*n* = 24 927) had a diagnosis of back problems, 8.8% (*n* = 5073) chronic pain, and 26.4% (*n* = 30 146) had no pain diagnosis. Pregabalin was prescribed the same day as an opioid to 38.1% of patients (95% confidence interval [CI] = 37.1% to 39.1%) and a benzodiazepine to 13.1% of patients (95% CI = 12.5% to 13.7%). Patients with a diagnosis of chronic pain had the highest rate of same-day prescribing of pregabalin with an opioid (70.4%, 95% CI = 68.9% to 71.9%) or a benzodiazepine (25.8%, 95% CI = 24.2% to 27.4%)

**Conclusion:**

Substantial increases in pregabalin prescribing were identified in Australian general practice, but only half of patients had a neuropathic pain diagnosis recorded, the only approved indication for subsidy. High rates of same-day prescribing with opioids and benzodiazepines may put patients at increased risk of harm.

## How this fits in

Pregabalin use and related harm have increased substantially in Australia since its public subsidy in 2013, but little is known about the conditions for which it is being prescribed. It was found that approximately half the patients prescribed pregabalin in general practice (between 2012 and 2018) had a recorded diagnosis of neuropathic pain during the study period, the only approved indication for subsidy, with many patients having recorded diagnoses of other pain conditions, such as back problems and chronic pain, for which pregabalin is not indicated. Same-day prescribing of pregabalin with opioids or benzodiazepines was common, despite the increased risks associated with these combinations. This study has identified subgroups of patients prescribed pregabalin who may be at increased risk of harm owing to potential off-label prescribing and co-prescribing with other sedative medicines.

## Introduction

Pregabalin is a gabapentinoid with anticonvulsant, analgesic, and anxiolytic properties. It is registered in Australia for the treatment of neuropathic pain and seizures, while it is also approved for generalised anxiety disorder in Europe and fibromyalgia in the US.^1^ In recent years, its use has increased markedly in North America, Europe, and the UK.^2–4^ In Australia, pregabalin dispensing nearly tripled in the first 4 years since it started to be subsidised in March 2013 by the PBS, the national programme that provides access to approved medicines.^5^ There are concerns it is being overprescribed,^2,6^ with several studies finding high rates of off-label use, particularly for non-neuropathic pain conditions where evidence of benefit is unclear.^2,3,7^

Pregabalin has abuse potential, and is commonly misused in people with a history of substance use disorder or psychiatric problems.^8–10^ When pregabalin is taken in combination with other medicines with sedative properties, particularly opioids and benzodiazepines, it can lead to central nervous system depression, with the greatest risk observed with high doses.^11^ In Australia, pregabalin recreational use, poisonings, and deaths have all been increasing,^5,11^ with a tenfold increase in pregabalin misuse-related ambulance attendances between 2012 and 2017.^9^ Pregabalin has also been associated with an increased risk of suicide.^12^ In the UK, pregabalin was reclassified to a controlled substance in 2019,^13^ and the Therapeutic Goods Administration (TGA), which regulates medicines in Australia, has been considering approaches to optimise safe use of pregabalin.^14^

While pregabalin is effective in reducing pain associated with certain types of neuropathic pain, such as diabetic neuropathy and postherpetic neuralgia,^15^ there is limited evidence for its efficacy for other types of pain, such as low back pain without neuropathic pain^16,17^ or with sciatica.^15,18^ Pregabalin is subsidised by the PBS solely for the treatment of neuropathic pain refractory to treatment by other medicines and not as a first-line therapy. However, previous work has identified that use of sub-therapeutic doses (25 mg) without up-titration is common, which is not consistent with treatment for neuropathic pain.^19^ To date, little is known about the conditions with which patients prescribed pregabalin have been diagnosed. The objective of this study was to describe patterns of pregabalin prescribing in Australian general practice, including: patterns of pregabalin prescribing over time; sociodemographic and clinical characteristics of patients prescribed pregabalin; and the prevalence of same-day prescribing of pregabalin with opioids and/or benzodiazepines.

## Method

### Study design and data source

A descriptive cross-sectional analysis was performed using Australian general practice clinical data from the MedicineInsight programme from 1 March 2012–28 February 2018. MedicineInsight collates de-identified electronic health records from the clinical information systems' best practice and medical director from consenting practices, which represents approximately 8% of Australian general practices.^20^ It is described in detail elsewhere.^21^ The data include patient demographic and clinical data recorded as part of routine clinical practice, including current and past prescribed medicines and diagnoses. Patients are assigned a unique identifying number at each practice site they attend allowing them to be followed in the database longitudinally over time.

### Study population

All patients aged ≥18 years were included at the 445 general practice sites, which met the data quality requirements at the time the MedicineInsight data were extracted for this study in May 2018.^21^ As some patients may have also visited general practices not contributing data to MedicineInsight, to maximise the probability of capturing relevant prescription and diagnostic information, a cohort of regularly attending patients ('active patients') was identified, who are likely to have received most of their care at participating practices. 'Active patients' were defined as those with ≥3 consultations in the previous 2 years at the same practice, in accordance with the Royal Australian College of General Practitioners' definition.^22^ Patients’ postcodes of residence were mapped to the Accessibility/Remoteness Index of Australia (ARIA+),^23^ and the Index of Relative Socioeconomic Advantage and Disadvantage (IRSAD).^24^

### Pregabalin prescriptions

Eligible Australian residents receive subsidised access to prescribed medicines listed with the PBS for the general population, or the Repatriation PBS (RPBS) for eligible veterans and their families. MedicineInsight captures PBS, RPBS, and private prescriptions. Private prescriptions can be written if the patient does not meet the specific PBS or RPBS subsidy requirements for prescribing a listed medicine and the patient pays the entire cost out-of-pocket. Prescription counts included all issued prescriptions.

Since 2005, pregabalin has been registered in Australia for treatment of neuropathic pain in adults, or adjunctive therapy in adults with partial seizures. Pregabalin has been subsidised for veterans through the RPBS since February 2008, and for the general population through the PBS from March 2013, solely for the treatment of neuropathic pain refractory to other medicines. It is neither registered nor subsidised for generalised anxiety disorder or fibromyalgia, for which it is approved in other jurisdictions. Pregabalin prescriptions were identified from the MedicineInsight data using the active ingredient obtained from the 'medicine name' and 'medicine active ingredient' fields.

### Prevalence of diagnoses and conditions

The recording of several relevant diagnoses were examined. This included neuropathic pain or sciatica, and epilepsy, which are indications approved by the TGA. Diagnoses of other pain conditions were identified, specifically back problems, a common off-label use of pregabalin,^16^ and unspecified chronic pain, a category that includes many types of pain. Depression was also identified, as it should be prescribed cautiously in people with depression, and mood changes are listed as potential adverse effects of pregabalin.^8,9,25^

In conjunction with medical, pharmacist, and clinical coding specialists, a search strategy was developed to identify conditions of interest from multiple data fields. While the data contain a 'reason for prescription' field, it is not completed for approximately 70% of prescriptions. As a result, the linking of recorded conditions directly with pregabalin prescribing could frequently not be done and so potential prescribing indications were identified using information from a combination of the 'reason for prescription', 'reason for encounter', and medical history (diagnosis) fields, including both coded (using Docle or Pyefinch codes) and free-text data. Diagnoses could be recorded at any time over the study period, including at a visit prior to prescribing of pregabalin, if pregabalin was a second-line therapy, or at a later visit, if the diagnosis was initially suspected and only later confirmed. A full list of included terms is in Supplementary Table 1. Patients could have multiple conditions recorded over the study period; if they had at least one diagnosis of neuropathic pain, they were counted in that group. To determine if neuropathic pain was adequately captured in the data, its recording in all active patients was also measured, not just those prescribed pregabalin.

### Same-day prescribing with opioids and benzodiazepines

Same-day prescribing of pregabalin with opioids and/or benzodiazepines was examined. A list of included medicines is in Supplementary Table 2. At the time of this analysis, only same-day prescribing in practices that used the best practice clinical information system for the whole study period could be examined owing to a technical issue, which represented 56.5% of patients prescribed pregabalin.

### Statistical analysis

As the number of active patients changes over time, to examine trends in pregabalin prescribing the proportion of all prescriptions that were for pregabalin per year were calculated, rather than counts or number of patients. The 95% confidence intervals (CIs) adjusted for practice clustering were calculated using PROC SURVEYFREQ and PROC SURVEYMEANS in SAS. Data analyses were conducted using SAS (version 9.4).

## Results

### Prescribing patterns

**Figure 1.**
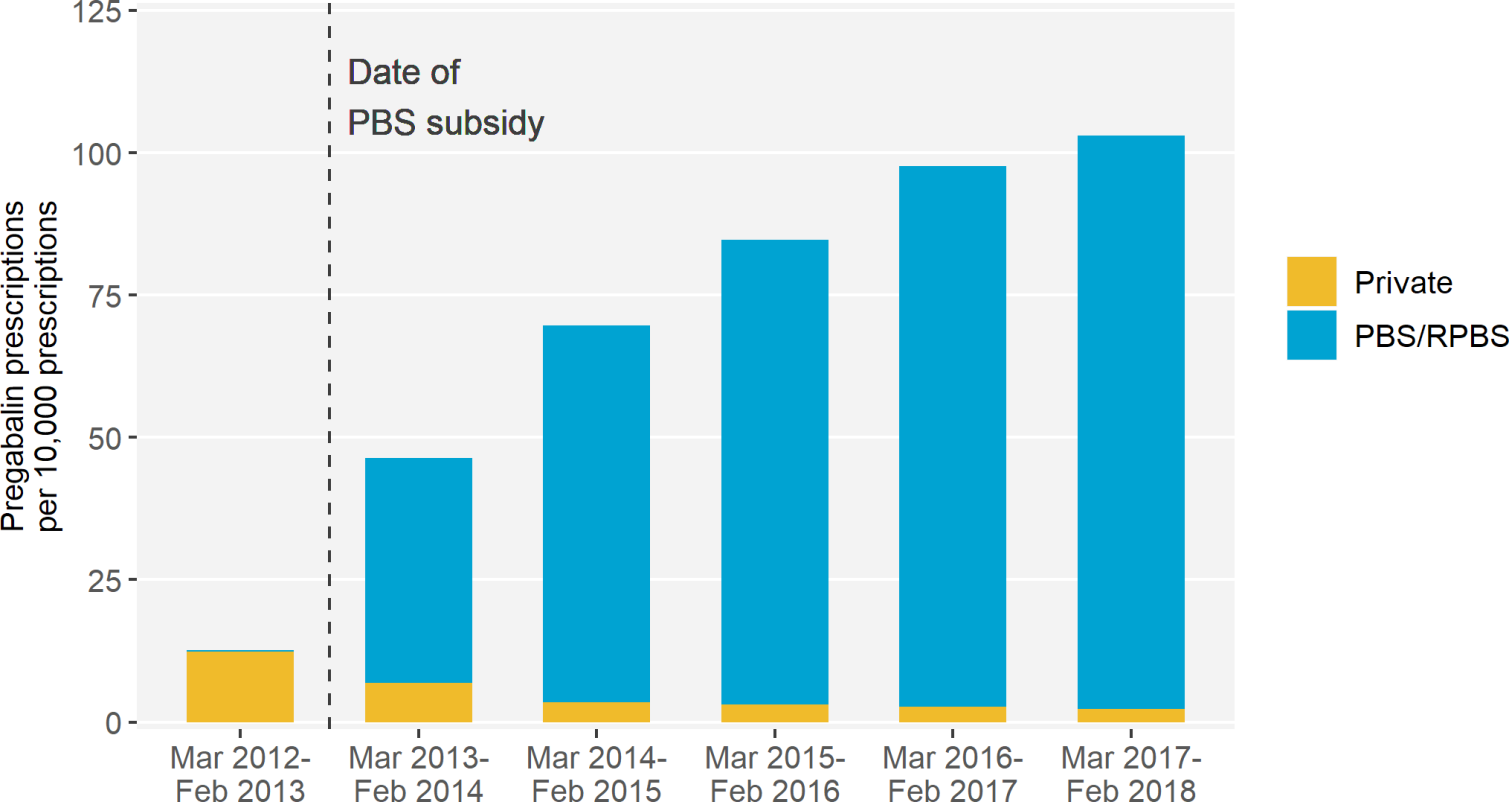
Pregabalin prescriptions as a proportion of all prescriptions by subsidy status. **Legend:** Excludes 6411 (1.6%) pregabalin prescriptions with missing prescription subsidy status. Prior to March 2013, pregabalin was only subsidised for veterans and their dependants. PBS = Pharmaceutical Benefits Scheme; RPBS = Repatriation Pharmaceutical Benefits Scheme.

Over the 6-year study period, there were 54 147 527 prescriptions of which 404 098 (0.8%) were for pregabalin. The rate of pregabalin prescribing increased from 13 per 10 000 prescriptions in the year prior to the PBS subsidy (March 2012–February 2013) to 104 per 10 000 prescriptions in year 5 post-subsidy (March 2017–February 2018) (Figure 1). Post-subsidy, most prescriptions were PBS or RPBS subsidised (95.8%). In the most recent year, only 2.2% were private prescriptions.

The 75 mg tablet was the most common strength prescribed. Prior to PBS subsidy, it represented 56.9% of all pregabalin prescriptions, decreasing to 42.9% of prescriptions in 2017–2018 (Supplementary Figure 1). Prescribing of higher strength tablets (150 mg and 300 mg) has remained relatively constant, while prescribing of the 25 mg tablet has increased, from 8.5% of prescriptions prior to the subsidy to 28.7% in 2017–2018.

### Characteristics of patients prescribed pregabalin

**Figure 2.**
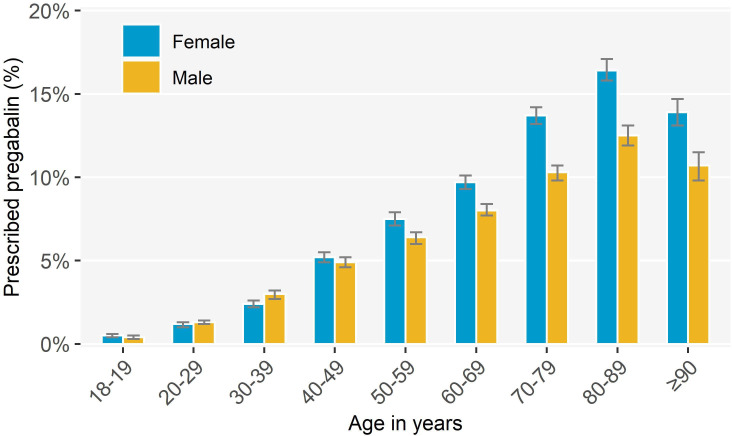
Proportion of active patients prescribed pregabalin by age group and sex, March 2012–February 2018 (*n* = 114 123).

A total of 1 891 623 active patients were identified (aged ≥18 years) of whom 114 123 (6.0%) had ≥1 pregabalin prescription. The median number of prescriptions per person was 1.4 (interquartile range, 1.0–3.8), and the mean was 3.5 (95% CI = 3.5 to 3.6). Pregabalin prescribing increased with age, with 14.7% (95% CI = 14.2% to 15.3%) of patients aged 80–89 years prescribed pregabalin (Figure 2 **;** Supplementary Table 3). Women aged ≥50 years were more likely to be prescribed pregabalin than men. Compared with the full study cohort, patients prescribed pregabalin were more likely to live in regional areas (that is, outside of major cities, excluding rural areas) and areas with more socioeconomic disadvantage (Table 1).

**Table 1.** Characteristics of the full study cohort and people prescribed pregabalin, March 2012–February 2018

	**All active patients (*n* = 1 891 623**)	**Patients prescribed pregabalin (*n* = 114 123**)
***n* (%**)	***n* (%**)	**Percentage of active patients, % (95% CI)^a^**
**Sex**			
Female	1 087 939 (57.5)	68 311 (59.9)	6.3 (5.9 to 6.6)
Male	800 676 (42.3)	45 769 (40.1)	5.7 (5.4 to 6.0)
Missing	3008 (0.2)	43 (0.0)	1.4 (0.8 to 2.0)
**Rurality of patient residence**			
Major city	1 204 426 (63.7)	63 456 (55.6)	5.3 (4.9 to 5.6)
Inner regional	440 727 (23.3)	33 852 (29.7)	7.7 (7.0 to 8.3)
Outer regional	200 543 (10.6)	14 706 (12.9)	7.3 (6.5 to 8.2)
Remote	29 386 (1.6)	1325 (1.2)	4.5 (3.2 to 5.8)
Very remote	5932 (0.3)	297 (0.3)	5.0 (3.4 to 6.6)
Missing	10 609 (0.6)	486 (0.4)	4.6 (3.9 to 5.3)
**Socioeconomic status of patient residence (IRSAD quintiles**)			
1 (most disadvantaged)	304 870 (16.1)	25 821 (22.6)	8.5 (7.9 to 9.1)
2	291 603 (15.4)	19 685 (17.2)	6.8 (6.3 to 7.2)
3	422 368 (22.3)	27 975 (24.5)	6.6 (6.1 to 7.1)
4	375 468 (19.8)	18 461 (16.2)	4.9 (4.5 to 5.3)
5 (least disadvantaged)	480 485 (25.4)	21 421 (18.8)	4.5 (4.0 to 4.9)
Missing	16 829 (0.9)	760 (0.7)	4.5 (3.8 to 5.2)

^a^Confidence intervals adjusted for clustering.

IRSAD index of relative socioeconomic advantage and disadvantage

In the full cohort, 6.1% (95% CI = 5.7% to 6.4%) had a diagnosis of neuropathic pain during the study period (Supplementary Table 4). Half of the patients prescribed pregabalin had a recorded diagnosis of neuropathic pain (including sciatica) (*n* = 56 772, 49.7%) (Table 2). Among patients without a recorded diagnosis of neuropathic pain, 43.5% (*n* = 24 927) had a diagnosis of a back problem and 8.8% (*n* = 5073) chronic pain. Throughout the study period, 30 146 patients (26.4%) had no recorded pain diagnosis. Only 1.0% (*n* = 1147) of patients had an epilepsy diagnosis, and 29.0% (*n* = 33 142) had a recorded diagnosis of depression.

**Table 2.** Pain diagnoses and conditions recorded during study period, March 2012–February 2018

	**Patients with a neuropathic pain^a^ diagnosis**	**Patients without a neuropathic pain^a^ diagnosis**	**Total**
	***n***	**%**	***n***	**%**	***n***	**%**
Total	56 772	100.0	57 351	100.0	114 123	100.0
Back problem^b^	34 406	60.6	24 927	43.5	59 333	52.0
Chronic pain^b^	6029	10.6	5073	8.8	11 102	9.7
Sciatica^b^	13 657	24.1	–	–	13 657	12.0
No neuropathic pain, back problem or chronic pain	–	–	30 146	52.6	30 146	26.4

^a^Includes sciatica. ^b^Individuals may have multiple diagnoses.

### Same-day prescribing with opioids and benzodiazepines

**Figure 3.**
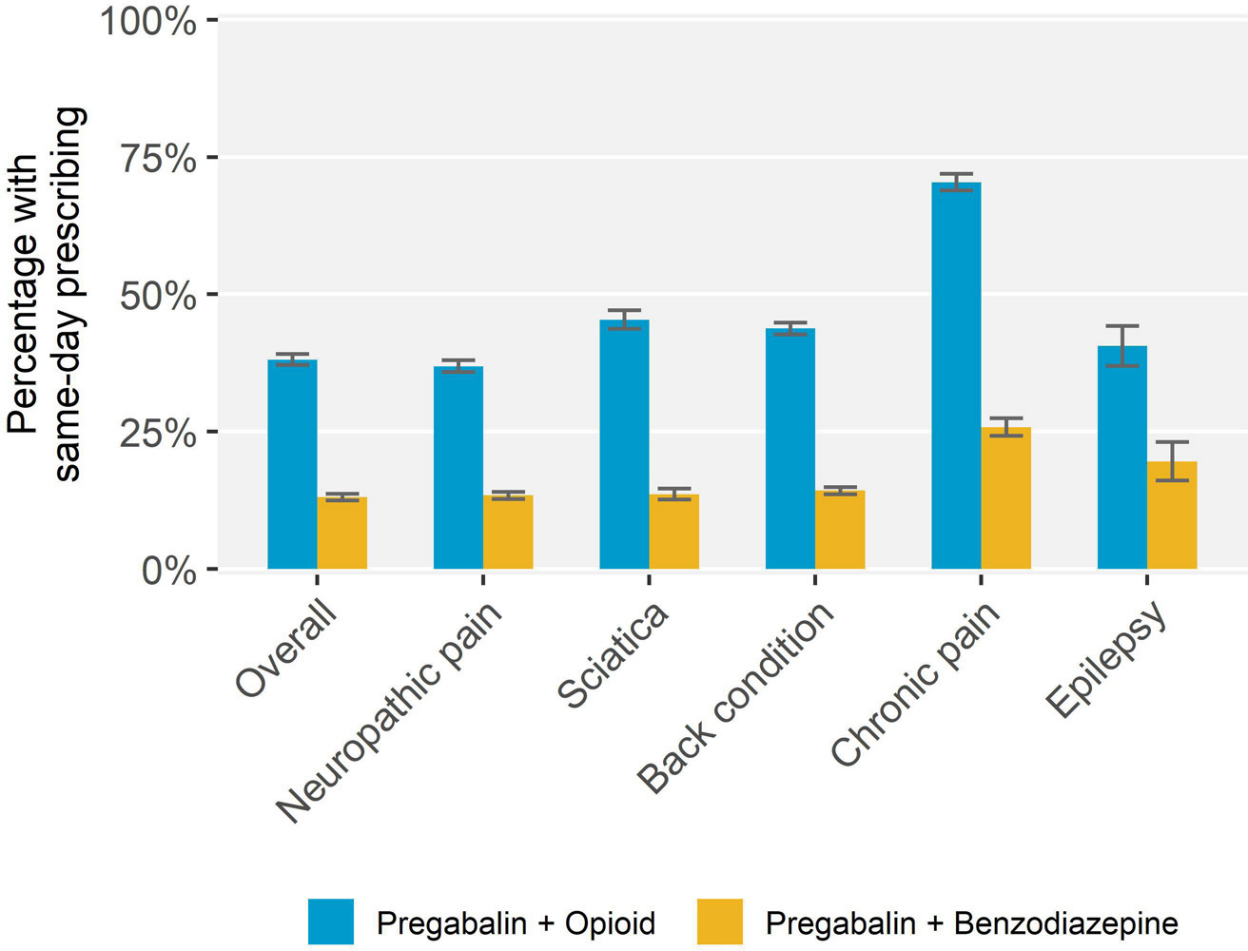
Prevalence of same-day prescribing of pregabalin and an opioid or benzodiazepine by recorded condition among patients prescribed pregabalin for whom capture of same-day prescribing was available, March 2012–February 2018 (*n* = 64 461). **Legend:** Conditions not mutually exclusive as patients could have multiple conditions.

The analysis of same-day prescribing included 64 461 active patients aged ≥18 years (56.5% of patients prescribed pregabalin). They had similar sociodemographic characteristics to the full cohort of patients prescribed pregabalin (Table 3). Pregabalin was prescribed on the same day as an opioid to 24 554 patients (38.1%, 95% CI = 37.1% to 39.1%), and the same day as a benzodiazepine to 8435 patients (13.1%, 95% CI = 12.5% to 13.7%); 4.4% (95% CI = 4.1% to 4.8%) were prescribed all three on the same day. Patients with a recorded diagnosis of chronic pain were most likely to have same-day prescribing of pregabalin and an opioid (*n* = 4732, 70.4%, 95% CI = 68.9% to 71.9%), or a benzodiazepine (*n* = 1733, 25.8%, 95% CI = 24.2% to 27.4%) than people with other diagnoses (Figure 3). Males and patients in the most disadvantaged areas were more likely to have same-day prescribing with either an opioid or benzodiazepine, and females were more likely to have same-day prescribing with a benzodiazepine, but these differences were small (Table 3).

**Table 3.** Characteristics of people with same-day prescribing of pregabalin and an opioid or benzodiazepine among people prescribed pregabalin for whom capture of same-day prescribing was available, March 2012–February 2018 (*n* = 64 461)

	**All active patients prescribed pregabalin, *n* (%**)	**Same-day prescribing of pregabalin and an opioid**	**Same-day prescribing of pregabalin and a benzodiazepine**
*****n*** (%**)	**Percentage of patients prescribed pregabalin, % (95% CI**)	***n* (%**)	**Percentage of patients prescribed pregabalin, % (95% CI**)
***n***	64 461 (100.0)	24 554 (100.0)	38.1 (37.1 to 39.1)	8435 (100.0)	13.1 (13.5 to 13.7)
**Sex**					
Female	39 058 (60.6)	14 376 (58.5)	36.8 (35.8 to 37.8)	5386 (63.9)	13.8 (13.2 to 14.4)
Male	25 380 (39.4)	10 170 (41.1)	40.1 (38.9 to 41.2)	3045 (36.1)	12.0 (11.3 to 12.7)
Missing	23 (0.0)	8 (0.0)	34.8 (13.0 to 56.6)	<5	
**Age group, years**					
18–19	132 (0.2)	36 (0.1)	26.9 (19.9 to 33.8)	8 (0.1)	6.0 (2.1 to 9.9)
20–29	2026 (3.1)	624 (2.5)	30.8 (28.4 to 33.2)	190 (2.3)	9.4 (7.9 to 10.9)
30–39	4899 (7.6)	1915 (7.8)	39.1 (37.0 to 41.2)	633 (7.5)	12.9 (11.7 to 14.2)
40–49	8760 (13.6)	3548 (14.4)	40.5 (39.0 to 42.0)	1191 (14.1)	13.6 (12.4 to 14.8)
50–59	11 834 (18.4)	4709 (19.2)	39.8 (38.5 to 41.1)	1539 (18.2)	13.0 (12.1 to 13.9)
60–69	13 912 (21.6)	5309 (21.6)	38.2 (36.9 to 39.4)	1745 (20.7)	12.5 (11.8 to 13.3)
70–79	13 548 (21.0)	4935 (20.1)	36.4 (35.3 to 37.6)	1732 (20.5)	12.8 (12.1 to 13.5)
80–89	7803 (12.1)	2904 (11.8)	37.2 (35.8 to 38.6)	1148 (13.6)	14.7 (13.7 to 15.8)
≥90	1545 (2.4)	574 (2.3)	37.2 (34.7 to 39.6)	249 (3.0)	16.1 (14.2 to 18.0)
**Socioeconomic status of patient residence (IRSAD quintiles**)					
1 (most disadvantaged)	11 437 (17.7)	4739 (19.3)	41.4 (39.7, 43.2)	1586 (18.8)	13.9 (12.9 to 14.9)
2	11 671 (18.1)	4629 (18.9)	39.7 (37.9, 41.4)	1536 (18.2)	13.2 (11.9 to 14.4)
3	16 833 (26.1)	6408 (26.1)	38.1 (36.4, 39.7)	2178 (25.8)	12.9 (11.9 to 14.0)
4	11 261 (17.5)	4167 (17.0)	37.0 (35.6, 38.4)	1451 (17.2)	12.9 (12.0 to 13.8)
5 (least disadvantaged)	12 851 (19.9)	4443 (18.1)	34.6 (32.7, 36.4)	1635 (19.4)	12.7 (11.6 to 13.8)
Missing	408 (0.6)	168 (0.7)	41.2 (35.1, 47.3)	49 (0.6)	12.0 (8.4 to 15.6)

IRSAD index of relative socioeconomic advantage and disadvantage

## Discussion

### Summary

This is the first study to describe the conditions with which patients prescribed pregabalin were diagnosed in Australian general practice. It was found that pregabalin prescribing increased eightfold over 6 years, with nearly one in seven patients aged 80–89 years receiving a prescription. Only half of the patients prescribed pregabalin had a neuropathic pain diagnosis recorded during the study period, even though it is the only PBS-approved indication, and over one-quarter had no recorded pain diagnosis. Same-day prescribing of pregabalin with opioids and/or benzodiazepines was high, despite the risks associated with the use of multiple sedative medicines.^10^ Lastly, increasing prescribing of the 25 mg tablet strength over the study period was observed, despite it being intended for dose titration only.

### Strengths and limitations

The MedicineInsight database provides national coverage of general practices, and the included patients are broadly representative of the Australian population.^21^ Unlike other national prescribing datasets in Australia, these data contain diagnoses recorded in general practice. The main limitations of these data are that the reason or indication for prescribing was not commonly recorded, and thus conditions could not be directly linked with pregabalin prescribing. Thus, other fields were relied on, such as medical history and reason for encounter, and conditions were identified over the entire study period to maximise the chance of identifying relevant diagnoses. This approach was necessary as neuropathic pain is typically a chronic condition lasting for years^26–28^ and clinicians may record the diagnosis in the medical history only once at initial diagnosis, even if it is managed for years. A study of pregabalin prescribing in UK general practice found that relying on diagnostic information from the day of prescribing severely underestimated the prevalence of neuropathic pain.^29^ However, not all recorded diagnoses of neuropathic pain were necessarily related to the prescribing of pregabalin. Additionally, some diagnoses may be missing as, for confidentiality reasons, MedicineInsight does not collect data from the unstructured area of the medical record contained in progress notes.

### Comparison with existing literature

The study identified 114 123 people prescribed pregabalin over the study period, representing 6% of all active patients, with increased prescribing of the 25 mg tablet strength. This is consistent with a 2020 Australian study that found that 5.3% of Australians initiated pregabalin over the same time period, including 15% of people aged ≥85 years, with high rates of dispensing of the 25 mg tablet strength without up-titration.^19^ While the study does not have information on prescribed dose, the 25 mg tablet is intended for dose titration purposes, and on its own it is not considered a therapeutic dose for neuropathic pain.^30^ In international studies, prescribing of pregabalin for unapproved or off-label indications is common.^2,7,29,31^ A 2019 study of pregabalin prescribing in UK general practice found that 55% of pregabalin prescriptions were off-label, primarily for non-neuropathic pain,^2^ while in a 2019 German study only 26% of people prescribed pregabalin had a neuropathic pain diagnosis.^7^ High rates of concomitant use of pregabalin, opioids, and benzodiazepines have also been observed in the US and the UK,^2,3^ and in patients initiating pregabalin, a majority had previously been prescribed or dispensed opioids.^4,19,31,32^

### Implications for research and practice

The study has identified substantial increases in pregabalin prescribing in Australian general practice over time. Approximately half of the patients had no definite diagnosis of neuropathic pain or epilepsy recorded, for which there are several potential explanations. First, neuropathic pain may be under recorded by GPs, or recorded with a non-specific term such as 'chronic pain'. However, the prevalence of neuropathic pain observed in the full cohort (6%) are similar to those observed in other Australian and international studies,^33,34^ and thus under recording may be minimal. Second, this may represent off-label prescribing, which is common in international studies. The increased prescribing of low strength tablets (25 mg) also points to prescribing for indications not well supported by evidence such as anxiety or insomnia,^15^ potentially putting patients at risk of unnecessary adverse effects such as somnolence, dizziness, and falls in older adults.^15,18,35^

A sciatica diagnosis was common in the present study's cohort (12%). In Australia, pregabalin is subsidised for neuropathic pain without any distinction for subtype and there is limited evidence of efficacy of pregabalin for sciatica,^15,18^ in contrast with other types of neuropathic pain such as postherpetic neuralgia or diabetic neuropathy.^15^ However, differentiating between pain syndromes is not straightforward, and finding effective treatment for chronic pain is challenging.^36,37^ High rates of prescribing of pregabalin combined with opioids or benzodiazepines in people with chronic pain were observed, despite the well-established risks.^10^ While there is little evidence of efficacy of opioids for treatment of neuropathic pain,^38^ pregabalin may sometimes be prescribed as adjunct therapy with opioids as it may reduce opioid consumption.^39^ Nevertheless, this combination should be used sparingly as it predisposes patients to harm, particularly in patients with pre-existing psychiatric problems or a history of substance use.^9,10^ In contrast, there are few situations where pregabalin should be prescribed with benzodiazepines.

The study has identified at-risk patient groups, owing to potential off-label prescribing and co-prescribing with other sedative medicines. However, the work also highlights the need for complete and accurate recording of diagnoses and reasons for prescribing in medical records, to better understand how and why medicines are being prescribed, both for clinical practice and research. This is especially important as Australia is currently moving toward greater use of electronic health records and primary care data for research.^40^ The TGA has recently advised changes to pregabalin’s product information and a targeted education strategy to increase prescriber awareness of the extent of pregabalin misuse.^14^ The aim of this is to increase awareness among prescribers of the potential harm associated with pregabalin, particularly in patients at high risk of adverse events such as people with depression, substance use disorders, or older people.
